# Diagnostic efficiency of RPA/RAA integrated CRISPR-Cas technique for COVID-19: A systematic review and meta-analysis

**DOI:** 10.1371/journal.pone.0276728

**Published:** 2022-10-26

**Authors:** Xiaoyu Zhang, Xiao Ge, Fangyuan Shen, Jinjuan Qiao, Yubo Zhang, Heng Li

**Affiliations:** Department of Medical Laboratory, Weifang Medical University, Weifang, Shandong, China; University of Helsinki: Helsingin Yliopisto, FINLAND

## Abstract

**Objective:**

To evaluate the diagnostic value of recombinase polymerase/ aided amplification (RPA/RAA) integrated clustered regularly interspaced short palindromic repeats (CRISPR) in the diagnosis of severe acute respiratory syndrome coronavirus 2 (SARS-CoV-2).

**Methods:**

We searched relevant literature on CRISPR technology for COVID-19 diagnosis using "novel coronavirus", "clustered regularly interspaced short palindromic repeats" and "RPA/RAA" as subject terms in PubMed, Cochrane, Web of Science, and Embase databases. Further, we performed a meta-analysis after screening the literature, quality assessment, and data extraction.

**Results:**

The pooled sensitivity, specificity and a rea under the summary receiver operator characteristic curve (AUC) were 0.98 [95% confidence interval (CI):0.97–0.99], 0.99 (95% CI: 0.97–1.00) and 1.00 (95% CI: 0.98–1.00), respectively. For CRISPR-associated (Cas) proteins-12, the sensitivity, specificity was 0.98 (95% CI: 0.96–1.00), 1.00 (95% CI: 0.99–1.00), respectively. For Cas13, the sensitivity and specificity were 0.99 (95% CI: 0.97–1.00) and 0.95 (95% CI: 0.91–1.00). The positive likelihood ratio (PLR) was 183.2 (95% CI: 28.8, 1166.8); the negative likelihood ratio (NLR) was 0.02 (95% CI: 0.01, 0.03).

**Conclusion:**

RPA/RAA integrated with CRISPR technology is used to diagnose coronavirus disease-19 (COVID-19) with high accuracy and can be used for large-scale population screening.

## 1 Introduction

The World Health Organization (WHO) declared COVID-19 a Public Health Emergency of international concern on January 30, 2020, and a pandemic on March 11, 2020 [1, 2]. COVID-19 is extremely harmful to the economy and public health of countries around the world, and we urgently need an efficient and accurate diagnostic method for large-scale population screening.

Real-time reverse transcriptase-polymerase chain reaction (RT-PCR) of nasopharyngeal swabs is commonly used to confirm the clinical diagnosis [3]. However, RT-PCR requires dedicated equipment and well-trained professional technicians, making them expensive and difficult to apply in areas with poor medical resources [4]. Therefore, it is particularly important to develop diagnostic test assays that are more rapid and easier to implement than RT-PCR. Recently developed Clustered Regularly Interspaced Short Palindromic Repeat (CRISPR) diagnostic technology has shown high sensitivity, specificity, rapidity, and convenience in pathogen technology [5, 6]. At present, the methods of combining recombinase polymerase/ aided amplification (RPA/RAA) with CRISPR/Cas systems (mostly Cas9, Cas12, and Cas13) have been used to detect SARS-CoV-2. We synthesized the relevant studies on the application of RPA/RAA integrated with CRISPR in the diagnosis of COVID-19 and evaluated its diagnostic value for COVID-19 to provide a theoretical basis for rapid large-scale screening of COVID-19.

## 2 Principle of CRISPR/Cas system

The CRISPR-Cas system establishes adaptive immunity in three stages: adaptation, expression, and interference [7]. When exogenous genes invade bacteria, bacteria cut exogenous genes into several spacer sequences and insert them into the bacterial genome. Guide RNAs encoded in CRISPR repeat region sequences are transcribed and processed into single CRISPR RNAs (crRNAs) that guide Cas proteins to recognize and cleave invading DNA or RNA in a sequence-dependent manner, which is the cis-cleavage activity of Cas proteins [8]. In addition, there is a trans-cleavage activity in Cas12 or Cas13 proteins, which can non-specifically cleave all single-stranded DNA (ssDNA) or single-stranded ribonucleic acid (ssRNA) in the system [9].

The complete genome of SARS-CoV-2 is about 30 kb, of which the first 2/3 close to the 5 ’end contain open reading frame 1ab (ORF1ab) genes encoding 16 non-structural proteins, and the last 1/3 contain genes encoding structural proteins including envelope protein (E), spike protein (S), nucleocapsid protein (N) and membrane protein (M) genes [10]. The cleavage activity of the Cas protein is provoked by exogenous delivery of crRNA that can specifically bind to the gene sequence in SARS-CoV-2. In the Cas12/Cas13-based systems, the presence of a cleavage reaction can be detected with a fluorescence reader or lateral flow strips by placing single-stranded DNA reporters labeled with fluorophore and biotin/quenching group [11, 12]; other Cas proteins can accurately detect the crRNA sequence of SARS-CoV-2 using their activity of targeted binding [13].

## 3 COVID-19 molecular diagnosis based on CRISPR/Cas system

### 3.1 CRISPR/Cas3 or Cas9 based diagnostics for COVID-19

Azhar *et al* [14] developed a method FELUDA (FnCas9 editor linked uniform detection assay) for the detection of SARS-CoV-2 based on the CRISPR-Cas9 system using FnCas9, a homolog of Cas9. FELUDA does not require trans-cleavage of reporter molecules, directly uses the digestion of Cas9 for nucleic acid detection, has a specificity and sensitivity of 98% and 96%, and is also highly accurate in detecting pathogenic single nucleotide variants and nucleic acid sequence analysis. Using the collateral single-stranded DNA cleavage activity of Cas3, Yoshimi *et al* developed a rapid (within 40 min), low-cost, instrument-free SARS-CoV-2 detection method, named CONAN [15] (Cas3-operated nucleic acid detection). CONAN not only detects SARS-CoV-2 in clinical samples but also offers specific detection of single-base-pair mutations in influenza virus A variants.

### 3.2 CRISPR/Cas12 based diagnostics for COVID-19

In 2020, based on the trans-cleavage of Cas12, Zhang Feng *et al* [16] developed STOP (SHERLOCK Testing in One Pot) for the detection of new-crowned viruses. STOP integrated sample processing, nucleic acid amplification, and detection in one tube, avoiding the effect of false positives caused by repeated uncapping. STOP is also combined with loop-mediated isothermal amplification technology (LAMP), which improves the sensitivity of the product, with viral loads as low as 10–100 copies/μL. Broughton *et al* [17] combined the previously established CRISPR-Cas12a-based targeting DETECTR (DNA endonuclease-targeted CRISPR trans reporter) and RT-LAMP technologies to detect SARS-CoV-2 N and E genes within forty minutes. The AIOD-CRISPR (all-in-one dual CRISPR-Cas12a) detection technology developed by Ding *et al* [18] uses a pair of Cas12a-crRNA complexes to bind different sites of the target sequence for specific recognition by targeting the nucleoprotein (N) gene of SARS-CoV-2. By adding a single-stranded DNA fluorophore-quencher (ssDNA-FQ) reporter, the detection results can be directly read by naked eyes in the background of blue LED lamp.

### 3.3 CRISPR/Cas13 based diagnostics for COVID-19

Jennifer Doudna *et al* [19] combined two different CRISPR enzymes, Cas13 and Csm6, to create a method that can detect a small amount of RNA virus within one hour, known as FIND-IT (Fast Integrated Nuclease Detection In Tandem), and this amplification-free technology provides a rapid and inexpensive diagnostic strategy for COVID-19. To address the need for reagents as well as expensive equipment, Rauch *et al* [20] developed CREST (Cas13-based, Rugged, Equitable, Scalable Testing) using the advantage of widely available enzymes (Cas13), low-cost thermocyclers (DIY-Bio and mini PCR/mini 16), and easy-to-use fluorescent visualizers, providing a bedside solution for COVID-19. The LOD of the CREST protocol was up to 10 copies of a target RNA molecule per microliter which unveiled that CREST is as sensitive as the corresponding RT-qPCR. At the same time, the authors also proposed PEARL [21] (Precipitation Enhanced Analyte Retrieval), which can avoid a convenient RNA extraction method using commercial kits, and the scalability of CREST can be further improved if it is used in combination.

We summarized various CRISPR/Cas based detection platforms (Table 1).

**Table 1:** CRISPR-based COVID-19 nucleic acid detection system.

platform name	Cas protein	Time (min)	Lod(copy/μL)	Target genes	Amplification system	References
**FELUDA**	Cas9	55	10	N、S	RT-PCR	[14]
**CONAN**	Cas3	40	1	N	RT-RPA	[15]
**STOP**	Cas12	50	100	N	RT-LAMP	[16]
**DETECTR**	Cas12	45	10	N	RT-LAMP	[17]
**AIOD-CRISPR**	Cas12	20	5	N	RT-RPA	[18]
**FIND-IT**	Cas13	20	31	N、S、ORF1ab	-	[19]
**CREST**	Cas13	120	10	N	PCR	[20]

## 4. Materials and methods

Article screening and data extraction in our work were followed by the proposal of the Preferred Reporting Items for Systematic Review and Meta-Analysis (PRISMA-P) 2009 (S1 Checklist).

### 4.1 Search strategy

We used "COVID-19" or "SARS-CoV-2" or "coronavirus disease-19" or " severe acute respiratory syndrome coronavirus 2" and "CRISPR" or "clustered regularly interspaced short palindromic repeats" as subject terms or keywords to search the literature published in PubMed, Embase, Cochrane Library, and Web of Science before April 2, 2022 (S1 File).

### 4.2 Study selection

All articles were screened according to the inclusion and exclusion criteria. The inclusion criteria were as follows: (1) The study subjects were suspected or confirmed COVID-19 cases; (2) both peer-reviewed and preprint original articles on RPA/RAA integrated CRISPR/Cas technology; (3) The purpose of the research is to evaluate the accuracy of CRISPR diagnostic method; (4) The extracted or calculated data could be used to obtain true-positive (TP), false-positive (FP), false-negative (FN), and true-negative (TN) values. The exclusion criteria were as follows: (1) Non-English Literature; (2) Non-Clinical research literature consisting of conference abstracts, reviews, and case reports; (3) studies based only on either RPA/RAA or CRISPR-Cas assays related to COVID-19.

### 4.3 Data extraction

We made a self-made scale to extract relevant information for inclusion in the study, including: (1) study first author;(2) year of publication;(3) TP, FP, FN, and TN;(4) location of study;(5) types of specimens;(6) targeted genes;(7) the type of Cas protein. When controversial results were encountered, they were negotiated by a third researcher.

### 4.4 Quality assessment

QUADAS-2 (Quality Assessment of Diagnostic Accuracy Studies 2) is currently the most recommended quality evaluation tool for diagnostic accuracy tests and is mainly composed of four parts: case selection, test to be evaluated, gold standard, case process and progress. Literature quality evaluation was performed by two investigators independently according to QUADAS-2 to evaluate the included literature, and when inconsistent evaluation was encountered, it was solved by negotiation.

### 4.5 Statistical analysis

The Spearman correlation coefficient and *I*^*2*^ test reflect the heterogeneity of threshold and non-threshold effects, respectively. We assessed the sensitivity, specificity, and diagnostic odds ratio (DOR), positive likelihood ratio (PLR), and negative likelihood ratio (NLP) of the 95% confidence interval (CIs) between RPA/RAA combined with CRISPR and the reference standard. We assessed diagnostic accuracy by summary receiver operating characteristic (SROC) curves and area under the curve (AUC), with high accuracy at AUC above 0.9. We used Deeks ’ funnel plot to determine whether this article had publication bias. The meta-analysis was undertaken using STATA17.0 and Meta-Disc 1.4. Quality assessments of included studies were carried out with RevMan 5.4 and SPSS 26. Differences with P < 0.05 were considered statistically significant.

## 5. Results

### 5.1 Study selection

**Fig 1:**
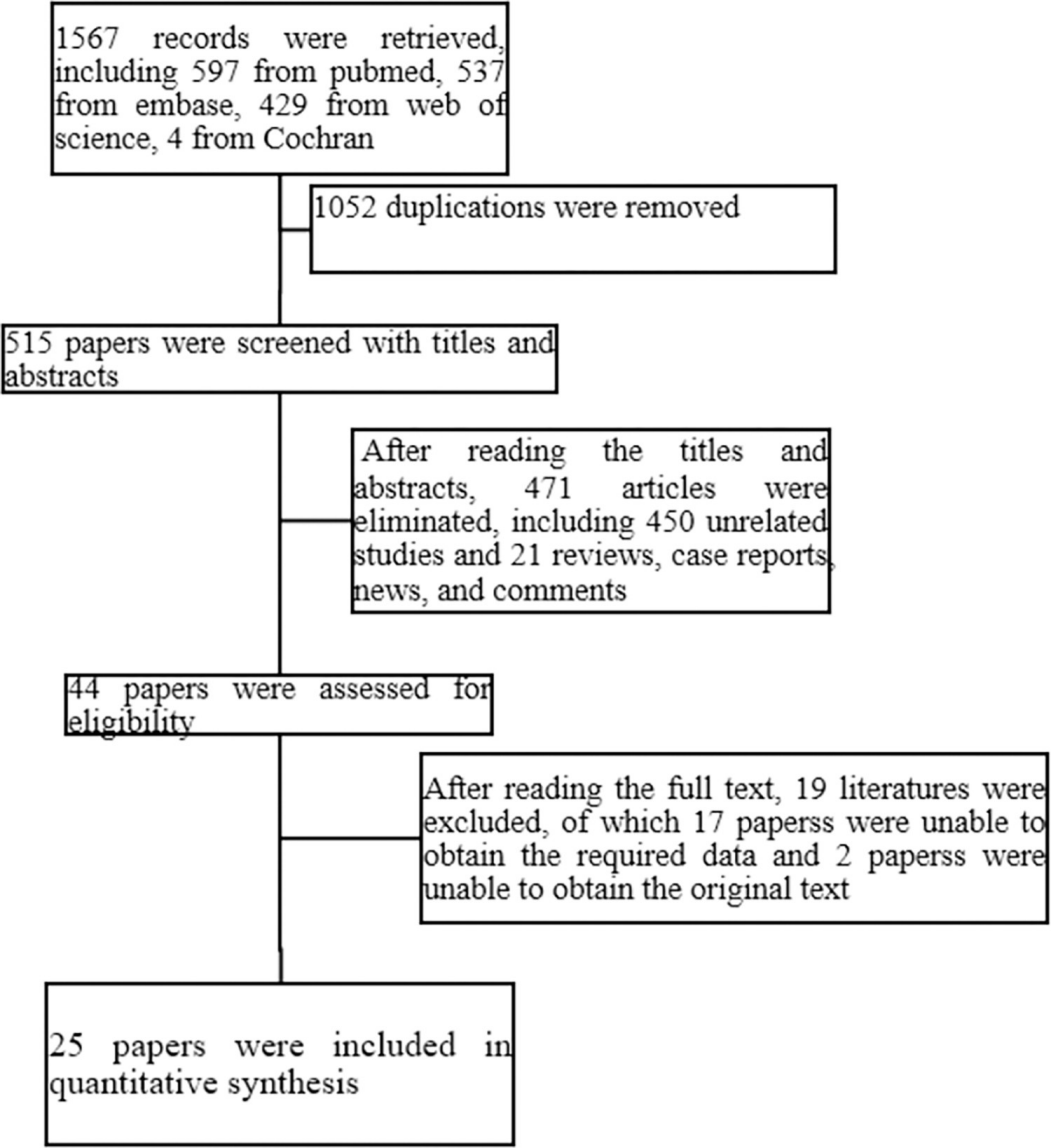
Flow diagram of the study selection process.

Following title, abstract, and full text screening according to inclusion and exclusion criteria, a final 25 articles were included for descriptive and numerical analysis in this scoping review, as outlined in the PRISMA flow diagram (Fig 1). The general information on the included studies is presented in Table 2.

**Table 2:** Major characteristics of included studies.

author	year	TP	FP	FN	TN	Cas	gene	Region	type	References
**Yin**	2021	8	0	0	13	Cas12	N、S	United States	NPS	[22]
**Ali**	2022	74	3	0	17	Cas9	orf1b	Saudi Arabia	NPS	[23]
**Patchsung**	2020	78	3	0	73	Cas13	S	Thailand	NPS/OPS	[24]
**Nimsamer**	2021	42	0	3	63	Cas12	N、S	Thailand	NPS/OPS	[25]
**Huang**	2021	2	0	0	7	Cas12	N	China	NPS/OPS	[26]
**Talwar**	2021	12	0	0	8	Cas12	S	Korea	nasal swabs/sputum	[27]
**Mayuramart**	2021	51	0	2	111	Cas12	S	Thailand	NPS/OPS	[28]
**Hou**	2020	52	0	0	62	Cas12	orf1ab	China	NPS/ bronchoalveolar lavage fluid specimens	[29]
**Tian**	2022	15	0	1	16	Cas12	O、N	China	NPS	[30]
**Lu**	2022	98	0	6	100	Cas12	orf1b、E	China	NPS	[31]
**Ma**	2020	13	0	0	11	Cas12	E	China	NPS	[32]
**Helena**	2021	26	1	1	20	Cas13	N	United States	saliva	[33]
**Sun**	2021	6	0	0	9	Cas12	N	China	swab samples	[34]
**Chen**	2021	11	0	0	16	Cas12	-	United States	NPS	[35]
**Azhar**	2021	14	0	1	32	Cas9	N、S	India	saliva/blood	[14]
**Wang**	2020	16	0	0	15	Cas12	E	China	-	[12]
**Tsou**	2021	10	0	0	12	Cas12	M、N	United States	NPS	[36]
**Xiong**	2020	11	0	0	11	Cas12	N	China	NPS	[37]
**Azmi**	2021	45	0	2	29	Cas13	S、N、E、RdRp	India	saliva	[38]
**Zhang**	2022	62	0	0	25	Cas13	N	China	-	[39]
**Li**	2021	243	25	3	378	Cas13	N	China	swab samples/sputum	[40]
**Erhu**	2021	34	1	0	29	Cas12	E、Orf1ab	China	NPS/saliva	[41]
**Ning**	2021	30	0	0	30	Cas12	N、E、Orf1ab	United States	NPS	[42]
**Ding**	2020	8	0	0	20	Cas9	E、Orf1ab	United States	nasal swab/saliva	[18]
**Marsic**	2021	54	2	0	4	Cas9	N	Saudi Arabia	nasal swab/saliva	[43]

TP: true-positive; FP: false-positive; FN: false-negative; TN: true-negative (TN); NPS: nasopharyngeal swab; OPS: oropharyngeal swab; orf1ab: open reading frame 1ab; RdRp: RNA-dependent RNA polymerase

### 5.2 Literature quality evaluation

Risk of bias assessment and applicability concerns for each study was carried out using the QUADAS-2 tool. In terms of case selection, because COVID-19 is more special and case selection has some limitations, studies have a high risk of bias; In terms of diagnostic tests to be evaluated, because there is no clear experimental design or unclear expression, some studies do not perform diagnosis by blind method, so the risk of bias is mostly unclear; In terms of reference standard and case process, real-time PCR was used for all reference standard tests, so the risk of study bias was a low risk of bias. In terms of applicability, except for case selection, which has some limitations, the other aspects have a high evaluation, so the overall quality of the literature included in the study is good.

### 5.3 Meta analysis

#### 5.3.1 Diagnostic threshold effect analysis and heterogeneity test

**Fig 2:**
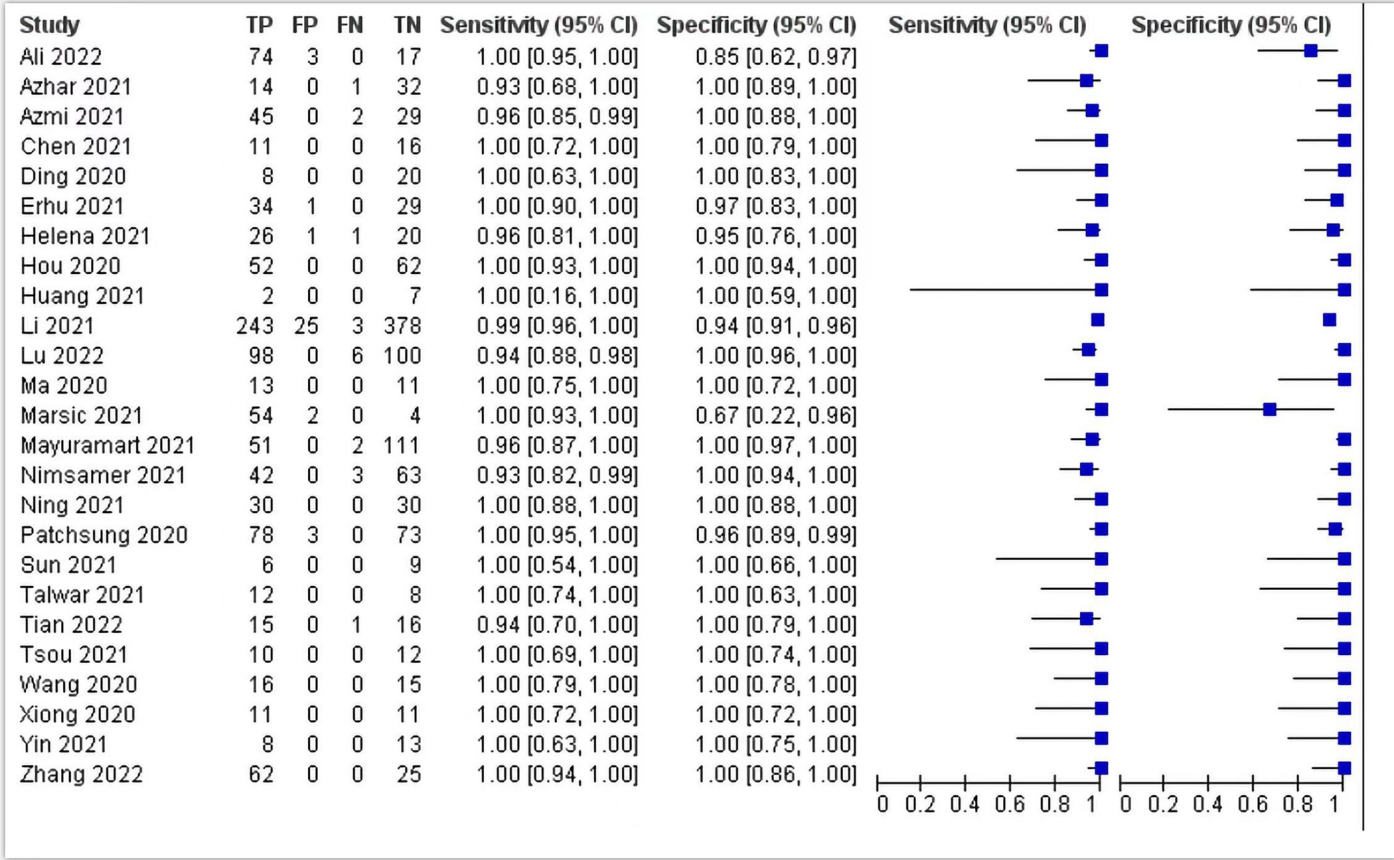
Forest plots of pooled sensitivity and specificity.

The results showed no heterogeneity caused by the threshold effect as the spearman correlation coefficient was 0.327 and the P value was 0.111 (> 0.05). However, it was observed that there was significant heterogeneity caused by non-threshold effects. The sensitivity of *I*^*2*^ was 42.54, and the specificity of *I*^*2*^ was 72.20, indicating that there was overall heterogeneity, but the sensitivity and specificity of most studies are close to 1 (Fig 2 and Table 3). And meta-regression was conducted to perform sources of heterogeneity.

**Table 3:** Pooled effect size.

Parameter	Estimate	95% *CI*	*I* ^*2*^
**Sensitivity**	0.98	(0.97, 0.99)	42.54
**Specificity**	0.99	(0.97, 1.00)	72.20
**Positive Likelihood Ratio**	183.2	(28.8, 1166.8)	-
**Negative Likelihood Ratio**	0.02	(0.01,0.03)	-
**Diagnostic Odds Ratio**	10702	(1765,64903)	-

#### 5.3.2 The diagnostic effect of CRISPR

**Fig 3:**
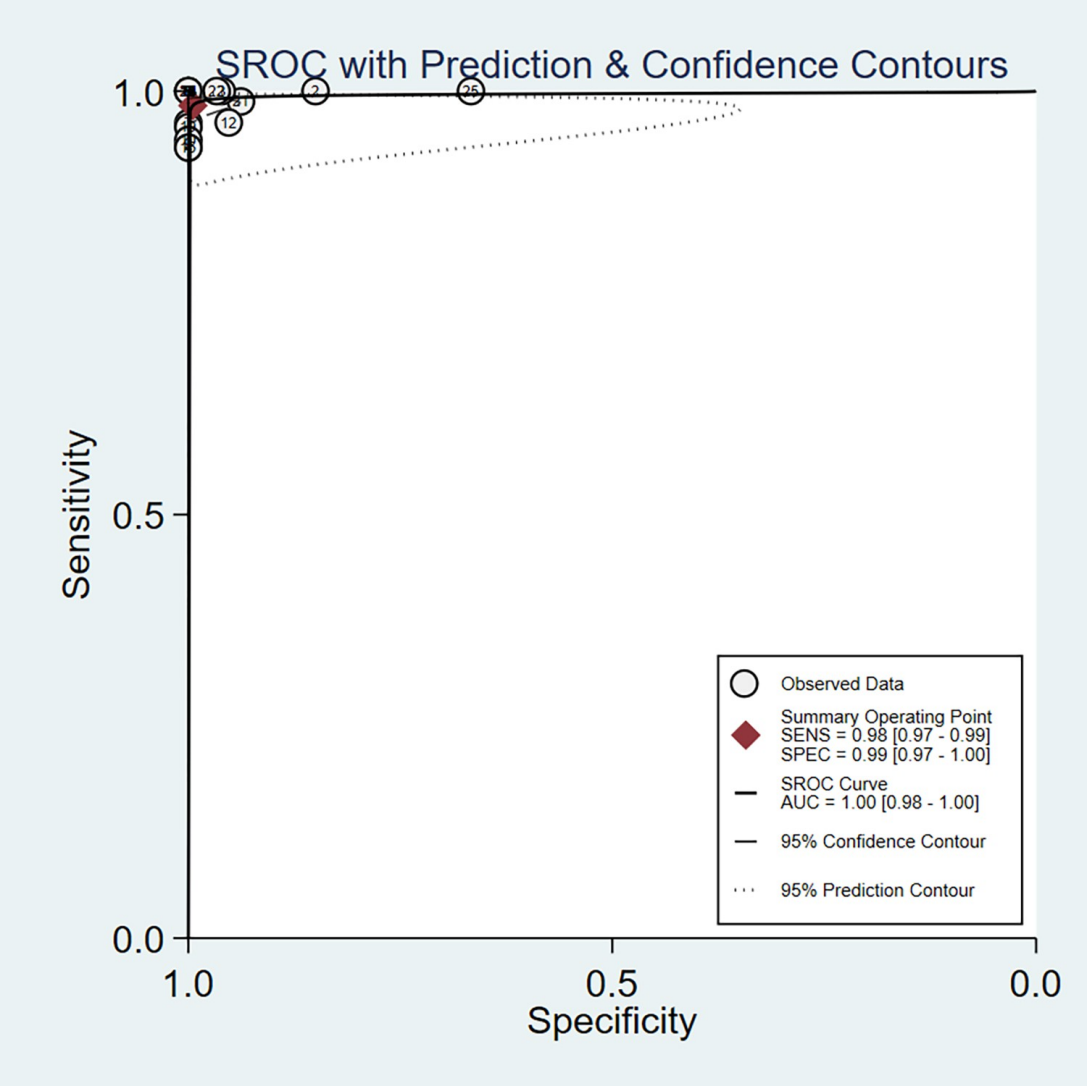
SROC curve of CRISPR for the diagnosis of COVID-19.

Diagnostic efficacy parameters for RPA/RAA combined with CRISPR were derived by pooling effect sizes. The pooled sensitivity, specificity, PLR, NLR and DOR were 0.98 (0.97,0.99), 0.99 (0.97,1.00), 183.2 (28.8,1166.8), 0.02 (0.01,0.03) and 10702 (1765,64903) (Table 3), respectively. In addition, the SROC curve showed that AUC was 1.00 (0.98–1.00) (Fig 3).

### 5.3.3 Subgroup analysis

We performed the meta-regression based on the variables including publication country, sample type, Cas type, and number of Genes, to explain this heterogeneity. Meta-regression analysis indicated that region and Cas type were the sources of heterogeneity for sensitivity and specificity. Additionally, the P value of the sample type was less than 0.05 indicating that it might be a significant source of heterogeneity in specificity (Table 4).

**Table 4:** Meta-regression.

Covariate	category	studies	Sensitivity	P1	Specificity	P2
**Cas**	Cas12	16	0.98 (0.96–1.00)	0.04	1.00 (0.99–1.00)	0.00
	Cas13	5	0.99 (0.97–1.00)		0.95 (0.91–1.00)	
**Gene**	single	14	0.99 (0.98–1.00)	0.20	0.98 (0.96–1.00)	0.89
	Multiple	10	0.96 (0.94–0.99)		1.00 (1.00–1.00)	
**Region**	Domestic	11	0.98 (0.97–1.00)	0.01	1.00 (0.99–1.00)	0.00
	Abroad	14	0.98 (0.97–1.00)		0.99 (0.98–1.00)	
**Sample type**	nasopharyngeal swab	9	0.98 (0.96–1.00)	0.99	1.00 (0.99–1.00)	0.00
	Other	5	0.97 (0.94–1.00)		0.99 (0.96–1.00)	

### 5.3.4 Sensitivity analysis and publication bias

**Fig 4:**
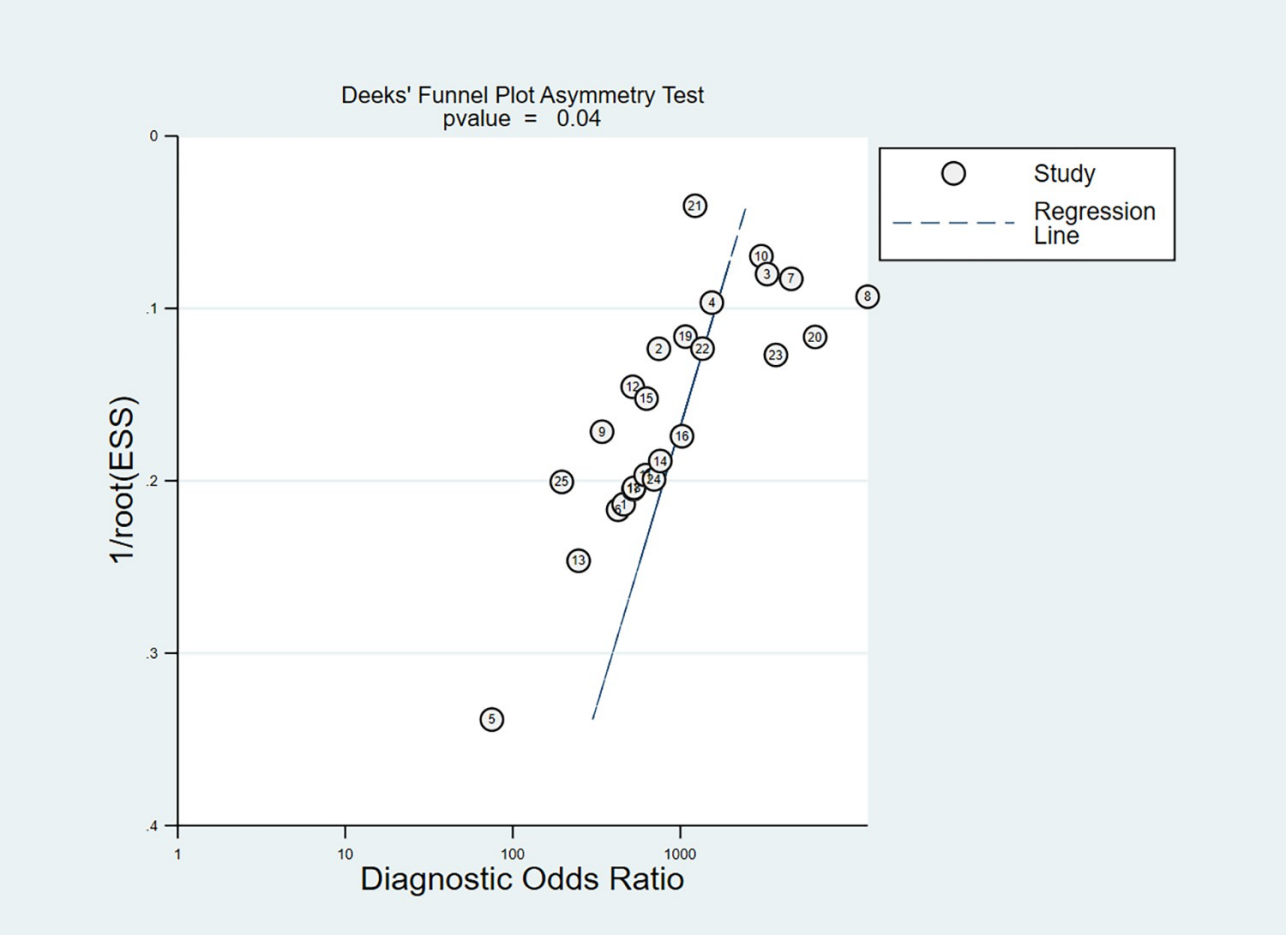
Deek’s funnel plot.

Individual articles were eliminated one by one, and then recalculated and analyzed to observe *I*^*2*^, P values, and the combined effect size and 95% CI after individual removal. The results suggest that a single article has little effect on the above indicators, indicating that the study results are stable. Deeks’ funnel plot was used to evaluate whether there was a publication bias in the included studies; P < 0.05 indicates significant publication bias (Fig 4).

## 6. Discussion

The results of this study indicate that the detection of COVID-19 by RPA/RAA integrated CRISPR technology is a technique with high diagnostic efficacy. In this meta-analysis, we have included only those articles which were solely based on RPA/RAA integrated CRISPR technique used for COVID-19 diagnosis. After the implementation of several inclusion and exclusion criteria as reported above, finally, 25 articles have been thoroughly assessed and selected for further analysis. Through QUADAS-2 analysis of the included studies, the quality of the articles generally performed well and more reliable results could be obtained.

Our included literature was performed using RT-qPCR as the gold standard, and RPA/RAA combined with CRISPR showed an accuracy of the approximate gold standard for the diagnosis of COVID-19. Whether CRISPR can be used as an alternative to RT-qPCR, we compared the two assays by self-scale (Table 5). We pooled RNA extraction approach and the CT values of RT-qPCR from 25 articles, and the CT values of positive specimens were not the same in each article, but all floated within the same range (< 40) and met the "Diagnosis and Treatment Protocol for Novel Coronavirus Pneumonia (Trial Eighth Edition)" criteria (S2 File). Factors such as disease severity, sample collection time, and extraction approach will cause differences in positive CT values, and 25 laboratories cannot ensure that the above factors are completely consistent, so positive CT values in the range of less than 40 are reasonable and will not have much impact on the gold standard RT-qPCR.

**Table 5:** RT-qPCR vs CRISPR in SARS-CoV-2.

Components	RPA/RAA with CRISPR	RT-qPCR
**Time**	<1h	2-4h
**Isothermal amplification**	Yes	No
**Multiple testing**	Yes	Yes
**Limit of detection (LoD)**	5–10 copies/μL	1 copy/μL
**Assay components**	Quantitative	Quantitative
**Bulky instrumentation required**	No	Yes
**Advantage**	Fast, easy, visual	High sensitivity and specificity, stable and reliable
**Disadvantage**	The research system and platform are not mature enough	Requires complex instruments and professionals
**Applied**	Suitable for screening in medically underdeveloped and remote areas	Clinical or center for disease control and prevention Laboratories

The pooled AUC of CRISPR in the diagnosis of COVID-19 was 1.00 (0.98–1.00). AUC is an important approach to assessing the accuracy of diagnostic tests, when the AUC is ≥ 0.9, it indicates an excellent diagnostic test [44]. Generally, a PLR higher than 10 indicates that the test under consideration is sufficient to confirm the diagnosis, while an NLR less than 0.10 means that the negative index test is sufficient to rule out the target disease. The pooled PLR and NLR were 183.2 (28.8,1166.8) and 0.02 (0.01,0.03), respectively. The range of DOR is from 0 to infinity, reflecting the connection between the results of diagnostic tests and diseases; a higher value suggests that the diagnostic test has a stronger discriminatory ability between patients and healthy people. The pooled DOR was 10702 (1765,64903). The results show that CRISPR has good diagnostic performance and high diagnostic value.

Meta-regression showed that the region and Cas type were the causes of heterogeneity in sensitivity and specificity (P < 0.05). Regarding regional classification, because the number of studies we included is not large enough, and it is only a simple classification of regions into domestic and foreign types, this result is not of great significance. Small but statistically significant differences (p < 0.05) were found for Cas type in terms of sensitivity and specificity. Sixteen studies used Cas12, five used Cas13, and four others used Cas9. Cas13 directly acts on the genetic material of coronaviruses, that is RNA, so the detection step is relatively simple and the detection time is shorter compared with Cas12. Both Cas12/Cas13 combined with RPA/RAA techniques show extremely high sensitivity and specificity in diagnosing COVID-19, and the same conclusion was made in Wang’s study [45]. In sample type analysis, to unify the criteria, we did not include the literature on multiple types of samples containing nasopharyngeal swabs in the analysis. We classified the literature on other sample types (saliva, blood, bronchoalveolar lavage fluid specimens, etc.) as the "Other" group. The specificity of a single nasopharyngeal swab was higher than the "Other" group by meta-regression analysis. The conclusion is supported by other studies that suggest that nasopharyngeal swabs have a higher diagnostic effect value and a better diagnostic effect than saliva, but given the difference in sampling time, we believe that saliva sampling is a reasonable alternative to nasopharyngeal swabs [46].

RPA/RAA is the commonly used amplification technique, but the pre-amplification process increases detection time, LAMP combined with CRISPR technology for the diagnosis of COVID-19 is another commonly used detection method [47], but compared with RPA/RAA, LAMP requires two pairs of primers and is more prone to false positives [48], so more and more unamplified CRISPR technologies are being developed for nucleic acid detection [49]. At present, amplification-free techniques are divided into the following three strategies [50]: (I) Reduce the reaction volume to increase the target concentration and improve the LOD (limit of detection); (II) Combined electrochemical biosensors; (III) Cas-mediated cascade amplification. We analyzed the amplification-free CRISPR technology for COVID-19, but due to the insufficient number of articles to combine effect sizes, the specificity and sensitivity of each study were simply extracted for discussion. The specificity of amplification-free CRISPR technology is high, but in Liang’s [51] study in terms of sensitivity, the detection sensitivity was as low as 0.66 and the performance was suboptimal. This shows that areas with high requirements for detection time can use amplification-free for initial screening and reduce time costs, but in terms of accuracy, amplification-free CRISPR technology still needs to continue to be optimized. We summarized the CRISPR/Cas-based amplification-free platforms (Table 6).

**Table 6:** Amplification-free CRISPR.

author	year	TP	FP	FN	TN	Sensitivity	Specificity	References
**Liang**	2021	21	0	11	80	0.66	1	[51]
**Li**	2021	35	0	17	80	0.67	1	[52]
**Li** ^**a**^	2021	38	0	14	80	0.73	1	[52]
**Lee**	2021	10	0	0	10	1	1	[53]
**Zhao**	2021	30	0	0	30	1	1	[54]

a: Multiple sets of valid data for the same article.

This study has three objective factors that warrant discussing: (I) the number of included studies is small, and although the use of the single literature elimination method to verify that single literature has little effect on the pooled effect size results, the more included studies, the sample size of the study subjects, the more persuasive the results; (II) different target genes, sample types, sampling methods, storage conditions, transportation methods may cause bias in the results, and these factors cannot be controlled; (III) our analysis indicated significant publication bias in included studies. Given that CRISPR technology is a newly developed nucleic acid detection technology and the detection applied to new crown viruses is also in the early development stage, many of the platforms developed based on the CRISPR system and evaluation of diagnostic accuracy for new crown viruses are performed by the same research group. Thus, there is likely to be bias toward reporting higher diagnosis performance.

## 7. Conclusion

The rapid spread of the COVID-19 pandemic has severely affected thousands of people’s health and has had a significant impact on the global economy. At present, although RT-qPCR can be used as the gold standard method for COVID-19 detection, due to the lack of reagents and equipment, COVID-19 cannot be found and diagnosed in a timely manner in areas with less developed medical conditions. The use of RPA/RAA integrated CRISPR-Cas in COVID-19 diagnosis has made a significant impact that should be endorsed for further optimization and improvement. Especially in resource-limited areas, patients can get their COVID-19 test reports in a short period, which is crucial in the event of an outbreak. Based on the results we analyzed, it can be concluded that RPA/RAA integrated CRISPR performs well and has great potential as an alternative to RT-qPCR for the diagnosis of COVID-19 in the resource-poor area.

## Supporting information

S1 Checklist Reporting items for systematic review and meta-analysis (PRISMA-P) 2009 statement guideline. (DOC)

S1 File Search strategy used for the systematic and meta analysis on diagnostic efficiency of RPA/RAA integrated CRISPR-Cas technique for COVID-19. (DOCX)

S2 File Values of 25 parameters for RT-qPCR. (DOCX)
