# Supplementary material for: Diagnostic efficiency of RPA/RAA integrated CRISPR-Cas technique for COVID-19: A systematic review and meta-analysis
Source: PLoS One. 2022 Oct 26;17(10):e0276728. doi: 10.1371/journal.pone.0276728 (PMC9604878; doi:10.1371/journal.pone.0276728)
Supplement: S1 File — (DOCX) [file pone.0276728.s002.docx]

# **Supplementary file 1: search strategy used for the systematic and Meta analysis on Diagnostic efficiency of RPA/RAA integrated CRISPR-Cas technique for COVID-19**

**PubMed:**((((CRISPR[Title/Abstract]) OR (CRISPR Sequences[Title/Abstract])) OR (Clustered Regularly Interspaced Short Palindromic Repeat[Title/Abstract])) OR (CRISPR Elements[Title/Abstract])) OR (CRISPR Loci[Title/Abstract])) OR (CRISPR Clusters[Title/Abstract])) OR (CRISPR Locus[Title/Abstract])) OR (CRISPR Arrays[Title/Abstract])) OR (CRISPR Spacers[Title/Abstract])) OR (CRISPR Spacer Sequences[Title/Abstract])) OR (CRISPR-Cas Loci[Title/Abstract])) OR (CRISPR-Cas Locus[Title/Abstract])) OR (CRISPR/Cas[Title/Abstract])) AND (((((((((((((((((((((((SARS-CoV-2 Infection[Title/Abstract] OR (SARS-CoV-2[Title/Abstract])) OR (SARS CoV 2 Infection[Title/Abstract])) OR (2019 Novel Coronavirus Infection[Title/Abstract])) OR (2019-nCoV Disease[Title/Abstract])) OR (COVID-19 Virus Infections[Title/Abstract])) OR (Coronavirus Disease 2019[Title/Abstract])) OR (Coronavirus Disease 19[Title/Abstract])) OR (Severe Acute Respiratory Syndrome Coronavirus 2 Infection[Title/Abstract])) OR (SARS Coronavirus 2 Infection[Title/Abstract])) OR (COVID-19 Virus Diseases[Title/Abstract])) OR (2019-nCoV Infections[Title/Abstract])) OR (COVID-19 Pandemic[Title/Abstract])) OR (SARS Coronavirus 2[Title/Abstract])) OR (Coronavirus Disease 2019 Virus[Title/Abstract])) OR (2019 Novel Coronaviruses[Title/Abstract])) OR (Wuhan Seafood Market Pneumonia Virus[Title/Abstract])) OR (SARS-CoV-2 Virus[Title/Abstract])) OR (SARS-CoV-2[Title/Abstract])) OR (COVID-19 Viruses[Title/Abstract])) OR (Wuhan Coronavirus[Title/Abstract])) OR (Severe Acute Respiratory Syndrome Coronavirus 2[Title/Abstract])) OR (COVID 19[Title/Abstract]))
