# Supplementary material for: Diagnostic efficiency of RPA/RAA integrated CRISPR-Cas technique for COVID-19: A systematic review and meta-analysis
Source: PLoS One. 2022 Oct 26;17(10):e0276728. doi: 10.1371/journal.pone.0276728 (PMC9604878; doi:10.1371/journal.pone.0276728)
Supplement: S2 File — (DOCX) [file pone.0276728.s003.docx]

**Table. Values of 25 parameters for RT-qPCR**

| **Author** | **Extraction Approach** | **Nucleic acid amplification kit/assay** | **Positive Samples CT Value** | **Cut off Value** |
| --- | --- | --- | --- | --- |
| **Yin** | QIAamp DSP viral RNA mini kit (Qiagen N.V., Venlo) | - | 16-29 | - |
| **Ali** | Quick Extract DNA extract solution (QE09050, Lucigen) | - | ≤36 | ≤36 |
| **Patchsung** | MagLEAD 12gC automated extraction platform (Precision System Science) | Allplex 2019-nCoV assay (Seegene) | 11-37 | ≤40 |
| **Nimsamer** | MagLEAD 12gC instrument | Allplex 2019-nCoV assay (Seegene) | ＜38 | ≤40 |
| **Huang** | CDC assay | RT-qPCR kit (BioGerm Medical Technology Co.) | - | ≤38 |
| **Talwar** | Quick Extract RNA solution | - | 14-30 | - |
| **Mayuramart** | GenUPTM Virus RNA kit (BiotechRabbit, German) | - | 10.5-34.7 | - |
| **Hou** | QIAamp Viral RNA Mini kit (Qiagen, Valencia, CA). | Novel Coronavirus (2019-nCoV) Real Time Multiplex RT-PCR Kit（Liferiver, Shanghai, China） | ＜40 | ＜40 |
| **Tian** | CFDA approved RT-qPCR kits | CFDA approved RT-qPCR kits. | 25.2-37.4 | ≤38 |
| **Lu** | according to the manufacturer’s protocol (liferiver). | - | 18.1-35.8 | - |
| **Ma** | QIAamp RNA Viral Kit (Qiagen, Heiden, Germany) | - | ＜38.9 | ＜40 |
| **Helena** | introduces 4 ml of saliva into the sample preparation chamber | Perkin Elmer | 14-38 | ≤42 |
| **Sun** | nucleic acid extraction kit (Da’An Gene., Ltd.) | RT-qPCR kit (BioGerm Medical Technology Co.) | ＜38.9 | ≤38 |
| **Chen** | unprocessed clinical NP swab eluates | CDC, USA | 18.3~30.2 | ＜40 |
| **Azhar** | - | STANDARD M nCoV Real-Time Detection kit (SD Biosensor) | ＜30 | ≤38 |
| **Wang** | QIAamp RNA Viral Kit (Qiagen, Heiden, Germany) | - | - | - |
| **Tsou** | - | SARS-CoV-2 test kit (Yaneng Biotech, Shenzhen,) | ≤35 | ＜40 |
| **Xiong** | RNA extraction kit (Health Biomed) | SARS-CoV-2 test kit (Yaneng Biotech, Shenzhen,) | ＜40 | ＜40 |
| **Azmi** | Qiagen viral RNA extraction kit | 360 Diagnostic and Health Services; | ＜35 | ＜40 |
| **Zhang** | automatic nucleic acid extractor (Jiangsu perBiotechnology Co., Ltd.) | RT-qPCR Kit (Sansure Biotech Inc) | 13-38 | ＜40 |
| **Li** | QIAamp Viral RNA Mini Kit (Cat No. 52906; QIAGEN, Hilden, Germany) | RT-qPCR kit (Da An Gene Co., Ltd., Sun Yat-sen University, Guangzhou, China) | <40 | ＜40 |
| **Erhu** | EZ1 AdvancedXL automatic rapid nucleic acid extractor (Qiagen, Germany) | RT-qPCR Kit (BioGerm Medical Technology Co., Ltd.)＆ RT-qPCR Kit (Sansure Biotech Inc.) | <40 | ≤38；≤40 |
| **Ning** | Quick DNA/RNA viral kit (Zymo; Hilden, Germany) | CDC, China | <40 | <40 |
| **Ding** | QIAamp DSP Viral RNA Mini Kit (QIAGEN. Venlo, The Netherlands). | CDC, USA | <40 | <40 |
| **Marsic** | Trizol method | One-step RT-qPCR kit (Invitrogen) | ≤38 | - |
